# Investigation of uncertainty in internal target volume definition for lung stereotactic body radiotherapy

**DOI:** 10.1007/s12194-023-00737-y

**Published:** 2023-09-15

**Authors:** Daiki Nakanishi, Masataka Oita, Jun-Ichi Fukunaga, Taka-Aki Hirose, Tadamasa Yoshitake, Motoharu Sasaki

**Affiliations:** 1 Division of Radiology, Department of Medical Technology, Kyushu University Hospital, 3-1-1, Maidashi Higashi-Ku, Fukuoka, 812-8582 Japan; 2 Graduate School of Interdisciplinary Science and Engineering in Health Systems, Okayama University, 3-1-1 Tsushima-Naka, Kita-Ku, Okayama, 700-8530 Japan; 3 Department of Clinical Radiology, Graduate School of Medical Sciences, Kyushu University, 3-1-1, Maidashi Higashi-Ku, Fukuoka, 812-8582 Japan; 4 Graduate School of Biomedical Sciences, Tokushima University, 3-18-15 Kuramoto-Cho, Tokushima, Tokushima 770-8503 Japan

**Keywords:** 4DCT, 3DCT, Internal target volume, EPID imaging, Stereotactic body radiotherapy, Lung cancer

## Abstract

This study evaluated the validity of internal target volumes (ITVs) defined by three- (3DCT) and four-dimensional computed tomography (4DCT), and subsequently compared them with actual movements during treatment. Five patients with upper lobe lung tumors were treated with stereotactic body radiotherapy (SBRT) at 48 Gy in four fractions. Planning 3DCT images were acquired with peak-exhale and peak-inhale breath-holds, and 4DCT images were acquired in the cine mode under free breathing. Cine images were acquired using an electronic portal imaging device during irradiation. Tumor coverage was evaluated based on the manner in which the peak-to-peak breathing amplitude on the planning CT covered the range of tumor motion (± 3 SD) during irradiation in the left–right, anteroposterior, and cranio-caudal (CC) directions. The mean tumor coverage of the 4DCT-based ITV was better than that of the 3DCT-based ITV in the CC direction. The internal margin should be considered when setting the irradiation field for 4DCT. The proposed 4DCT-based ITV can be used as an efficient approach in free-breathing SBRT for upper-lobe tumors of the lung because its coverage is superior to that of 3DCT.

**Novel scientific points**

- Attempt to evaluate the validity of the ITVs defined by 3DCT and 4DCT in lung SBRT.
- The coverage of the motion range during irradiation for the ITV based on 4DCT was superior to that of 3DCT.
- The validity of the ITV-PTV margin was investigated based on respiration-induced tumor motion.

## Introduction

Lung cancer is the most common cause of death in men worldwide, surpassing all other cancers [1]. Non-small cell lung cancer (NSCLC) accounts for 85% of all lung cancers [2]. Treatment of NSCLC is determined based on the disease stage, histological type, age, and complications, and radiotherapy is selected if necessary [3, 4]. In recent years, stereotactic body radiotherapy (SBRT) has been used to treat NSCLC and has been reported to exhibit a high local control rate of 90% and a significant reduction in the occurrence of adverse events in normal tissues compared with conventional irradiation methods [5–10]. Treatment planning for SBRT in lung cancer requires accurate and precise target settings. However, owing to the movement of tumors caused by breathing, accurately defining the target is challenging.

According to the International Commission on Radiation Units and Measurements Report 62, the internal target volume (ITV) must be set by adding an internal margin that considers tumor movement to the clinical target volume [11]. For conventional treatment planning of SBRT under free breathing based on three-dimensional computed tomography (3DCT), end-of-exhale and end-of-inhale breath-hold images are used to yield an ITV [12–14]. Setting the ITV at the end-of-exhale and end-of-inhale breath-holds is simple and can be used in all CT systems. However, two-phase ITV based on 3DCT does not include the motion information of the intermediate phases. Therefore, the curved motion pathway during each breathing cycle may introduce uncertainty in the two-phase approach.

Four-dimensional computed tomography (4DCT) can depict the tumor in all respiratory phases, and the ITV can include the entire range where the tumor is present under free-breathing conditions [15–18]. The ITV generated using 4DCT has been shown to depict actual tumor motion more accurately than that generated using conventional 3DCT; however, few studies have compared the accuracy of ITV with tumor motion during treatment under free-breathing conditions [16, 19]. Recently, a study of real-time tumor motion monitoring during irradiation in magnetic resonance imaging (MRI)-guided lung SBRT reported that targeting should consider changes in both intra- and inter-fractional respiratory amplitudes [20]. To calculate the systematic and random errors in the internal motion of the tumor, cine images captured during radiotherapy using an electronic portal imaging device (EPID) can be used [21, 22]. Their study depended only on beams with gantry angles of 0° or 180°. However, this study used 3DCT and 4DCT for treatment planning and EPID cine images from various gantry angles during treatment to estimate the tumor center of gravity in the left-right (LR), antero-posterior (AP), and cranio-caudal (CC) directions. This study evaluated the validity of the ITVs defined using 3DCT and 4DCT for lung SBRT under free-breathing conditions. Subsequently, the ITVs were compared with actual tumor movements. If tumor motion is better depicted on 4DCT than on 3DCT compared with actual irradiation, 4DCT can be used for more accurate ITV settings.

## Methods and materials

### Patients

This retrospective study was approved by the Institutional Review Board of our hospital. Five patients (age, 77–86 years; mean age, 80 years) with NSCLC who underwent SBRT between February 2017 and April 2019 were included in the study. The male:female ratio was 3:2. Table 1 summarizes patient characteristics. The mean gross tumor volume (GTV) from the exhale-phase 3DCT was 3.0 ± 0.9 cm^3^. At our hospital, tumors with a 3D migration of ≤ 10 mm are irradiated under free-breathing conditions. This study investigated the ITV settings for irradiation under free respiration, and all patients had tumors in the upper lobe with relatively small respiratory migration.

**Table 1:** Patient characteristics used in this study

Patient	Tumor Location	GTV volume	Dose prescription	Gantry angle	Irradiation time	Imaging data
		(cm^3^)	(Gy/fractions)	(°)	(s)	
1	LUL	1.95	48/4	1st. 158.4	15.80	39 images
				2nd. 130.0	17.12	42 images
2	RUL	3.63	48/4	1st. 180.0	14.88	37 images
				2nd. 230.0	14.92	37 images
3	LUL	3.97	48/4	1st. 180.0	13.40	33 images
				2nd. 140.0	14.32	35 images
4	LUL	2.22	48/4	1st. 165.0	8.44	21 images
				2nd. 95.0	18.76	0 images
5	RUL	3.01	48/4	1st. 195.0	12.88	32 images
				2nd. 230.0	13.76	34 images

### CT simulation

In all patients, a 3DCT scan of the thoracic region was performed in the axial mode, followed by a 4DCT scan using an Aquilion PRIME CT scanner (Canon Medical Systems Co., Tochigi, Japan) with a breath track (Engineering System Co., Ltd, Nagano, Japan) that acquired displacements of a marker on the patient's abdomen using a charge-coupled device camera. A thermoplastic sheet (Engineering System Co., Ltd., Nagano, Japan) was used as an abdominal compression device to limit the motion of the tumor to < 10 mm in the 3D vector. 3DCT scans were acquired with a 2.0 mm slice thickness under end-of-exhale and end-of-inhale breath-holds. During acquisition, the waveform of the breath-track system was used to visually confirm that the breath was held firmly.

In our hospital, to depict the entire movement of the tumor per respiratory cycle, 4DCT scans were acquired only around an area that included the lung tumor during un-coached free breathing in the axial cine mode. The cine duration was set to 10.5 s, which was sufficiently longer than the normal respiratory cycle (4 s). Furthermore, the slice thickness was 0.5 mm, and the reconstructed time interval was 0.5 s, which generated 21-phase images. The CT data were reconstructed in a field of view of 500 mm on a 512 × 512 grid for both CT scans. The measurement of tumor motion for 3DCT and 4DCT was performed using the contouring software in Eclipse version 13.6 (Varian Medical Systems, Palo Alto, CA, USA). The software created a tumor structure for each respiratory phase and analyzed the shift in the center of gravity from the reference structure in all three directions.

### Treatment planning

Radiation treatment planning, including the delineation of the GTV on the CT image, was performed using Eclipse. The following two approaches were used to define the ITVs: (1) the GTVs were outlined on end-of-exhale and end-of-inhale breath-hold images of 3DCT, which were combined to form a 3DCT-based ITV, and (2) the GTVs were outlined on each of the 21 phases of 4DCT, which were combined to form a 4DCT-based ITV. Here, 3DCT- and 4DCT-based ITV envelope the GTV of 3DCT and 4DCT, respectively. Tumor regions extracted by different radiation oncologists have been reported to differ by up to 3 mm, and this uncertainty and a setup margin of 5 mm are included in the planning target volume (PTV) margin (8 mm) [23].

Treatment planning was performed using eight non-coplanar static photon beams of 6 MV flattening filter-free (FFF) and a 2 mm multi-leaf collimator margin with a total prescribed dose of 48 Gy to 95% of the PTV in four fractions.

### Image acquisition

Free-breathing cone-beam computed tomography (CBCT) was performed before irradiation to align the tumor with the isocenter. Initially, automatic registration of the bone anatomy was completed between the planning CT (end-of-exhale breath-hold 3DCT) and CBCT images, followed by manual refinement of the tumor location. To confirm the tumor location, EPID images (EPID 1st and EPID 2nd) were acquired sequentially using the first and second beams of each fraction under free-breathing conditions. Images of each patient were acquired using 6 MV FFF X-rays from a linear accelerator (TrueBeam STx, Varian Medical Systems, Palo Alto, CA, USA) in cine mode on an EPID (aS-1200, Varian Medical Systems). Using the cine mode of the EPID in this system, images were acquired every 0.04 s (image acquisition rate: 25 frame/s). During image acquisition, the source-to-axis distance (SAD) and source-to-imaging distance (SID) were set to 100 and 150 cm, respectively. The matrix size of the images was 1,024 × 768 pixels, and the pixel size was 0.392 mm. Forty image sets were acquired in four sessions for five cases, each consisting of images captured from two directions.

### EPID analysis

#### Target position reconstruction

**Fig. 1:**
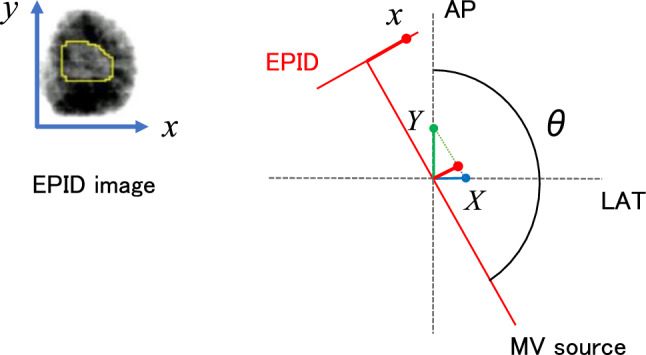
Relationship between the tumor position on the isocenter (X and Y) and its projection position x on an EPID image for an irradiation gantry angle θ. EPID, electronic portal imaging device

The tumor position on an EPID image was defined as the center of gravity of the tumor area, which was manually determined by one radiation oncologist and two radiologists using image processing software (Image J, National Institutes of Health, USA). The relationship between the tumor position on the isocenter (*X*, *Y*, and *Z*) and its projection position (*x* and *y*) on an EPID image for an irradiation gantry angle *θ* can be defined using the following equations:

1 $$X = x\cdot \frac{1}{\mathrm{cos}(180-\theta )}\cdot \frac{100}{150} (90\le \theta \le 180)\, or \,X = x\cdot \frac{1}{\mathrm{cos}\left(\theta -180\right)}\cdot \frac{100}{150} (180\le \theta \le 270),$$

2 $$Y = x\cdot \frac{1}{\mathrm{sin}(180-\theta )}\cdot \frac{100}{150} (90\le \theta \le 180)\, or \,Y = x\cdot \frac{1}{\mathrm{sin}\left(\theta -180\right)}\cdot \frac{100}{150} (180\le \theta \le 270),$$

3 $$Z = y\cdot \frac{100}{150}.$$

where an SAD of 100 cm is the distance between the MV sources, and an SID of 150 cm is the distance between the MV sources and the EPID (Fig. 1).

For each of the 20 treatment fractions, we calculated the magnitude of tumor motion in the 2D projection images. The irradiation gantry angles are listed in Table 1. The couch angle was set to 0°. In this case, there was no uncertainty in the motion in the CC direction, because the motion was perpendicular to the imaging beam at all gantry angles. However, the motion parallel to the beam cannot be resolved for motion in the LR and AP directions, and none of its moving components can be determined. Therefore, *X* and *Y* estimated the amount of movement using calculations. Because the projection images were acquired in two directions per fraction, calculations were performed for 40 treatment beam angles. Setup errors were obtained by averaging the residual tumor misalignment from the isocenter measured during irradiation in all patients.

### Tumor coverage

In radiotherapy for tumors with respiratory movements, the ITV must cover the actual movement of the tumor while setting an irradiation field. Therefore, the movement range coverage during irradiation of the ITV and PTV sets during planning was calculated. This study used three standard deviations (± 3 SD) from the mean position of the tumor on the EPID images as the range of breathing motion during irradiation. For each patient, the SD for inter-observer variations was calculated from the root mean square (RMS) of the random errors in tumor motion by the observers, as follows:

4 $$SD = \sqrt{\frac{1}{N}\sum_{j=1}^{N}{{\sigma }_{j}}^{2}}.$$

where *N* is the number of observers. σ_*j*_ represents the random error of tumor motion for an observer *j*.σ_*j*_ was given by

5 $${\sigma }_{j} = \sqrt{\frac{1}{n}\sum_{i=1}^{n}{{\sigma }_{i,j}}^{2}}.$$

where *n* is the number of image sets obtained using the EPID. σ_*i*,*j*_ represents the random error on an image set *i* for an observer *j*.

Tumor coverage was evaluated based on the extent to which the peak-to-peak breathing amplitude on the planning CT scan covered the range of tumor motion.

6 $$Tumor\ coverage = -(1-\frac{the\ peak-to-peak \, breathing \,amplitude \, on \, the \, planning \, CT}{6\,SD\,(tumor \,position \, on \, EPID \, images)})\times 100.$$

Using this equation, we evaluated whether the moving tumor was within the planned ITV during irradiation. PTV coverage was calculated by adding a PTV margin of 8 mm to the planned ITV. Here, the greater the planned ITV or PTV relative to the tumor motion during irradiation, the greater the tumor coverage. A value with a negative sign indicates that the tumor is outside the planned ITV or PTV.

### Statistical analysis

Statistical analyses were performed using JMP software version 16 (SAS Institute Inc., Cary, NC, USA). A paired t-test was used to determine whether 3DCT and 4DCT data were significantly different. Differences of *P* < 0.05 were considered statistically significant.

## Results

### Planning CT

Table 2 shows tumor motion and ITV volume on 3DCT and 4DCT. Tumor motion in the CC direction on 4DCT was significantly larger than that on 3DCT (paired t-test, *P* = 0.0256). However, the difference in tumor motion between the two groups was not significant in the LR (*P* = 0.1260) or AP (*P* = 0.5019) direction. Further, the mean 3D tumor motion vector was 5.0 and 2.7 mm for 4DCT and 3DCT, respectively, with a statistically significant difference (*P* = 0.0403). On 3DCT, the tumor motion in the AP direction was greater than that in either the LR (*P* = 0.6504) or CC (*P* = 0.6230) directions. However, for 4DCT, the tumor motion in the CC direction was greater than that in the LR (*P* = 0.5287) or AP (*P* = 0.0614). The ITV volume on 4DCT was larger than that on 3DCT (*P* = 0.0832).

**Table 2:** Tumor motion and ITV volume for 3DCT and 4DCT

Patient			1	2	3	4	5	Median (range)	Mean ± SD
3DCT	COM motion (mm)	LR	1.1	0.5	0.1	2.8	1.3	1.1 (0.1-2.8)	1.2 ± 1.0
		AP	1.4	0.9	3.7	1.7	0.2	1.4 (0.2-3.7)	1.6 ± 1.3
		CC	1.9	1.6	0.3	0.0	1.7	1.6 (0.0-1.9)	1.1 ± 0.9
		3D	2.6	1.9	3.7	3.3	2.1	2.6 (1.9-3.7)	2.7 ± 0.8
	ITV volume (cm^3^)		2.5	4.0	5.2	2.9	4.0	4.0 (2.5-5.2)	3.7 ± 1.1
4DCT	COM motion (mm)	LR	4.7	1.3	3.4	1.7	3.0	3.0 (1.3-4.7)	2.8 ± 1.4
		AP	1.4	1.6	2.1	2.5	2.8	2.1 (1.4-2.8)	2.1 ± 0.6
		CC	3.4	2.1	2.1	3.7	5.6	3.4 (2.1-5.6)	3.4 ± 1.4
		3D	6.0	2.9	4.5	4.8	6.9	4.8 (2.9-6.9)	5.0 ± 1.5
	ITV volume (cm^3^)		3.0	4.6	5.9	3.2	6.5	4.6 (3.0-6.5)	4.6 ± 1.6

### Tumor motion for each patient

**Fig. 2:**
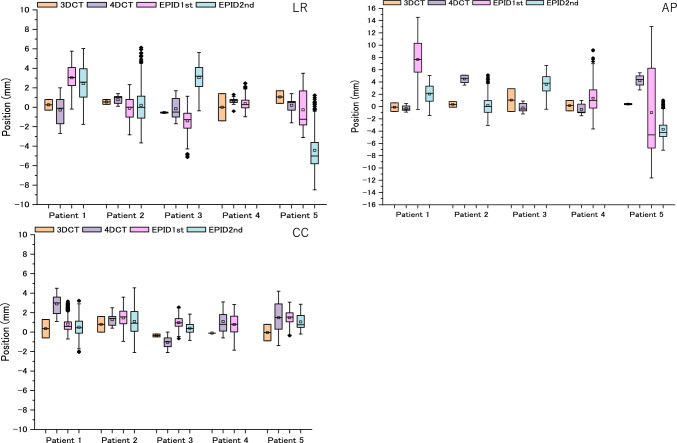
Tumor position assessed from 3DCT, 4DCT, and EPID for five patients. The centerline of each box and the square mark indicate the median and mean values, respectively. The lower and upper edges represent 25th and 75th percentiles, respectively. *3DCT,* three-dimensional computed tomography; *4DCT,* four-dimensional computed tomography; *EPID,* electronic portal imaging device.

The average setup errors in the LR, AP, and CC directions were 0.2, 1.1, and 0.9 mm, respectively. Table 3 presents the results of tumor motion during irradiation obtained by EPID analysis for all five cases. The standard deviations in the LR and AP directions were larger than those in the CC direction. Figure 2 shows the tumor center positions assessed using 3DCT, 4DCT, and EPID in the five patients. For EPID 1st in the AP direction in patients 2 and 3, the tumor location could not be theoretically calculated because the gantry angle was 180°. In addition, there were no data for EPID 2nd in patient 4 because the tumor and spine overlapped on the beam’s-eye-view image, and the tumor could not be extracted. Table 4 summarizes the tumor coverages for the ITV and PTV based on the 3DCT and 4DCT results. A value with a negative sign indicates that the tumor is outside the planned ITV or PTV. The 4DCT-based ITV exhibited better coverage of tumor motion measured during treatment than 3DCT, acquired under peak-exhale and peak-inhale breath-holds. In the major motion direction (i.e., the CC direction), the coverage of the 4DCT-based ITV and PTV was significantly larger than that of the 3DCT-based ITV and PTV (paired *t*-test, *P* = 0.0445). However, for the LR and AP directions, no significant difference was observed between the coverage of 3DCT-based ITV and PTV and 4DCT-based ITV and PTV (*P* = 0.2305 and *P* = 0.3913, respectively).

**Table 3:** The range of breathing motion in the LR, AP, and CC directions during irradiation

Patient		1	2	3	4	5
Standard deviation (mm)	LR	1.1	0.7	1.0	0.4	0.5
	AP	1.9	0.7	1.5	1.7	1.2
	CC	0.6	0.7	0.5	0.6	0.4

**Table 4:** Range-of-motion coverage during irradiation for ITV and PTV based on 3DCT and 4DCT

Patient			1	2	3	4	5	Mean ± SD
Coverage for ITV (%)	LR	3DCT	ｰ83.3	ｰ88.1	ｰ98.3	16.7	ｰ56.7	-62.0 ± 46.6
		4DCT	ｰ28.8	ｰ69.0	ｰ43.3	ｰ29.2	0.0	ｰ34.1 ± 25.1
	AP	3DCT	ｰ87.7	ｰ78.6	ｰ58.9	ｰ83.3	ｰ97.2	ｰ81.1 ± 14.2
		4DCT	ｰ87.7	ｰ61.9	ｰ76.7	ｰ75.5	ｰ61.1	ｰ72.6 ± 11.2
	CC	3DCT	-47.2	ｰ61.9	ｰ90.0	ｰ100.0	ｰ29.2	ｰ65.7 ± 29.4
		4DCT	ｰ5.6	ｰ50.0	ｰ30.0	2.8	133.3	10.1 ± 71.9
Coverage for PTV (%)		3DCT	159.1	292.9	168.3	683.3	476.7	356.1 ± 223.4
		4DCT	213.6	311.9	223.3	637.5	533.3	383.9 ± 191.4
	AP	3DCT	52.6	302.4	118.9	73.5	125.0	134.5 ± 98.7
		4DCT	52.6	319.0	101.1	81.4	161.1	143.1 ± 106.1
	CC	3DCT	397.2	319.0	443.3	344.4	637.5	428.3 ± 126.4
		4DCT	438.9	331.0	503.3	447.2	800.0	504.1 ± 176.8

## Discussion

In this study, we first analyzed the variation in the 3D motion of an upper lung tumor using 3DCT and 4DCT. The motion on 4DCT was significantly larger than that on 3DCT only in the CC direction (*P* = 0.0256). Seppenwoolde et al. reported that tumor motion in the upper lung lobe was smaller in the LR and AP directions than in the CC direction [24]. In this study, the amount of tumor movement in the CC direction was greater than those in the LR (*P* = 0.5287) and AP directions (*P* = 0.0614) in the 4DCT simulation. Thus, in the CC direction, where the amount of tumor movement was large, the tumor position changed over a wider range on 4DCT than on 3DCT.

Several studies have examined differences in the volume created by focusing on the number of phases used to create an ITV based on 4DCT [25–27]. Ezhil et al. showed that two-phase ITV (mean ± SD: 13.93 ± 15.69 cm^3^) were consistently smaller than all-phase ITV (mean ± SD: 16.60 ± 17.05 cm^3^) regardless of the magnitude of the CC motion [25]. Jang et al. examined the applicability of target volume definition based on two extreme phases of 4DCT data in SBRT planning for 17 patients with lung cancer. The results showed that in seven patients with 3D tumor motion of less than 1 cm, the overlap between the two- and all-phase ITV was 87.4 ± 3.9%, and that between the two- and all-phase PTV was 92.1 ± 2.5% [26]. Koksal et al. reported the mean overlap volume between two- and four-phase ITV and all-phase ITV as 89.4 ± 5.3% and 94.2 ± 2.4%, respectively, for nine patients with upper lung cancer [27]. Our results were comparable because we found that the ITV volume on 3DCT was 80% of that on 4DCT. Thus, using the two extreme phases of respiratory motion resulted in underestimation when setting the ITV, and the addition of motion information between the two extreme phases was more appropriate for determining the ITV.

In SBRT, preserving critical structures while administering an ablative dose in a clinical setting is challenging. In this case, the PTV coverage may be compromised, whereas the ITV coverage is not. Therefore, accurate ITV delineation is clinically important. In this study, no difference in the mean ITV coverage between 4DCT and 3DCT was observed in either the LR or AP direction. In contrast, in the CC direction, the range of motion of the tumor depicted by 4DCT was significantly larger than that depicted by 3DCT, and the ITV coverage set by 4DCT was also significantly larger. Using 4DCT, the ITV can be set more accurately in the CC direction, which is the main direction of movement for upper lung tumors. Thus, 4DCT-based ITV can be used as an efficient approach in free-breathing SBRT for upper lobe tumors of the lung because its coverage in the CC direction is superior to that of 3DCT.

The ITV underestimation of the actual tumor motion in 4DCT and 3DCT may be because random motion (error) during irradiation was not well captured by the planning CT. In addition, the low ITV coverage in the LR and AP directions was affected by the uncertainty in evaluating the motion. Although these random errors must be added as margins when setting the irradiation field, a PTV margin of 8 mm was isotropically set for the ITV and the irradiation field covered the tumor movements. Ueda et al. evaluated the position of the tumor center in the CC direction based on motion information obtained from planning 4DCT and EPID cine images acquired during irradiation for non-respiratory-gated radiotherapy [28]. They reported that the tumor motion on planning 4DCT was underrepresented compared with EPID cine images, with a margin of 6.0 and 8.0 mm to compensate for the uncertainty of the ITV at the cranial and caudal sides, respectively. Ge et al. reported that abdominal tumor motion during a 4DCT scan did not adequately represent tumor motion during treatment, particularly in patients undergoing SBRT [29]. They acquired the tumor motion during treatment using fluoroscopy. We believe that a margin is required to compensate for the uncertainty of the ITV created by the planning CT. In our study, a greater margin was required for 3DCT than for 4DCT, particularly in the CC direction.

Considering the results of Ueda et al., the PTV margin of 8 mm on both the cranial and caudal sides in this study was slightly larger than their set of 6.0 and 8.0 mm on the cranial and caudal sides, respectively; however, it is reasonable to refer to the PTV coverage [28]. A margin is required to prevent missing the target irradiation and unnecessarily irradiating a high proportion of normal tissue. Based on our finding that the PTV margin was sufficient to cover the actual tumor motion, the proposed conventional method was considered clinically acceptable. Because the ITV coverage of 4DCT was better than that of 3DCT, the internal margin for setting an irradiation field confined to the tumor can be considered while avoiding the normal tissue by utilizing 4DCT.

This study had certain limitations. First, the EPID cine images were acquired in two directions for each patient; however, they were acquired in one direction at a time and not simultaneously. Therefore, the actual amount of tumor movement in the LR and AP directions could not be determined. In this study, we estimated the movement of the tumor in the LR and AP directions at the isocenter during irradiation, based on the amount of movement of the tumor projected from one direction. The tumor localization accuracy of 2D projection imaging methods for 3D tumor motion has been previously reported. Suh et al. found that the magnitude of geometric uncertainty for 2D projections along the imaging beam axis of 3D tumor motion was approximately 2 mm [30]. Yip et al. reported that tumor-tracking errors were dependent on the gantry angles of beam's-eye-view (BEV) imaging during lung SBRT delivery [31]. They found that the error was approximately 2 mm for the BEV acquired with gantry angles of approximately 180°. We estimated the maximum values that could be obtained in the LR and AP directions, which would have resulted in an overestimation of the actual motion in these directions. This overestimation may be partly attributed to other organs such as the heart and mediastinum surrounding the upper-lobe tumors, thereby providing poor contrast in the EPID images. Subsequently, we evaluated whether the ITV set in the planning CT scans covered this movement.

Next, the ITV coverage obtained in this study was based on the ITV and the extent of tumor movement during irradiation. The average setup errors in the LR, AP, and CC directions were 0.2, 1.1, and 0.9 mm, respectively. Those in the AP and CC directions were slightly larger than those in the LR direction. This result is consistent with that of a study by Liang et al., who quantified tumor motion during irradiation based on treatment monitoring using orthogonal kV X-ray imaging in CyberKnife treatments [32]. The setup error in the three directions was less than the setup margin of 5 mm in this study; thus, the PTV sufficiently covered the actual range of tumor movement. Investigating the optimal ITV-PTV margin considering setup errors is a future issue.

To date, 4DCT-based planning has been shown to depict the ITV more accurately than conventional 3DCT-based planning; however, its accuracy has rarely been compared with that of tumor motion during treatment under free-breathing conditions. This study compared the tumor motion on planning 3DCT and 4DCT with that during treatment using EPID cine images. We conclude that 4DCT is superior to 3DCT in terms of ITV coverage.

## Conclusions

This study employed traditionally used target settings based only on end expiration and end aspiration for free-breathing SBRT in lung cancer. Furthermore, 4DCT was used to confirm the target volume, which showed that the range of tumor movement on 4DCT was larger than that on 3DCT in the CC direction. This is consistent with the movement observed during irradiation. Therefore, the tumor motion observed on 4DCT should be considered when setting the irradiation field in clinical practice. In future, it will be desirable to investigate the internal margin based on 4DCT, which can avoid the irradiation of normal tissues and confine irradiation to tumors.
